# Supplementary material for: The geography of COVID-19 spread in Italy and implications for the relaxation of confinement measures
Source: Nat Commun. 2020 Aug 26;11:4264. doi: 10.1038/s41467-020-18050-2 (PMC7449964; doi:10.1038/s41467-020-18050-2)
Supplement: Supplementary file 1 — Supplementary Information [file 41467_2020_18050_MOESM1_ESM.pdf]

The geography of COVID-19 spread in Italy and implications for the relaxation of confinement measures

Bertuzzo et al.

Supplementary Information

Supplementary Figures

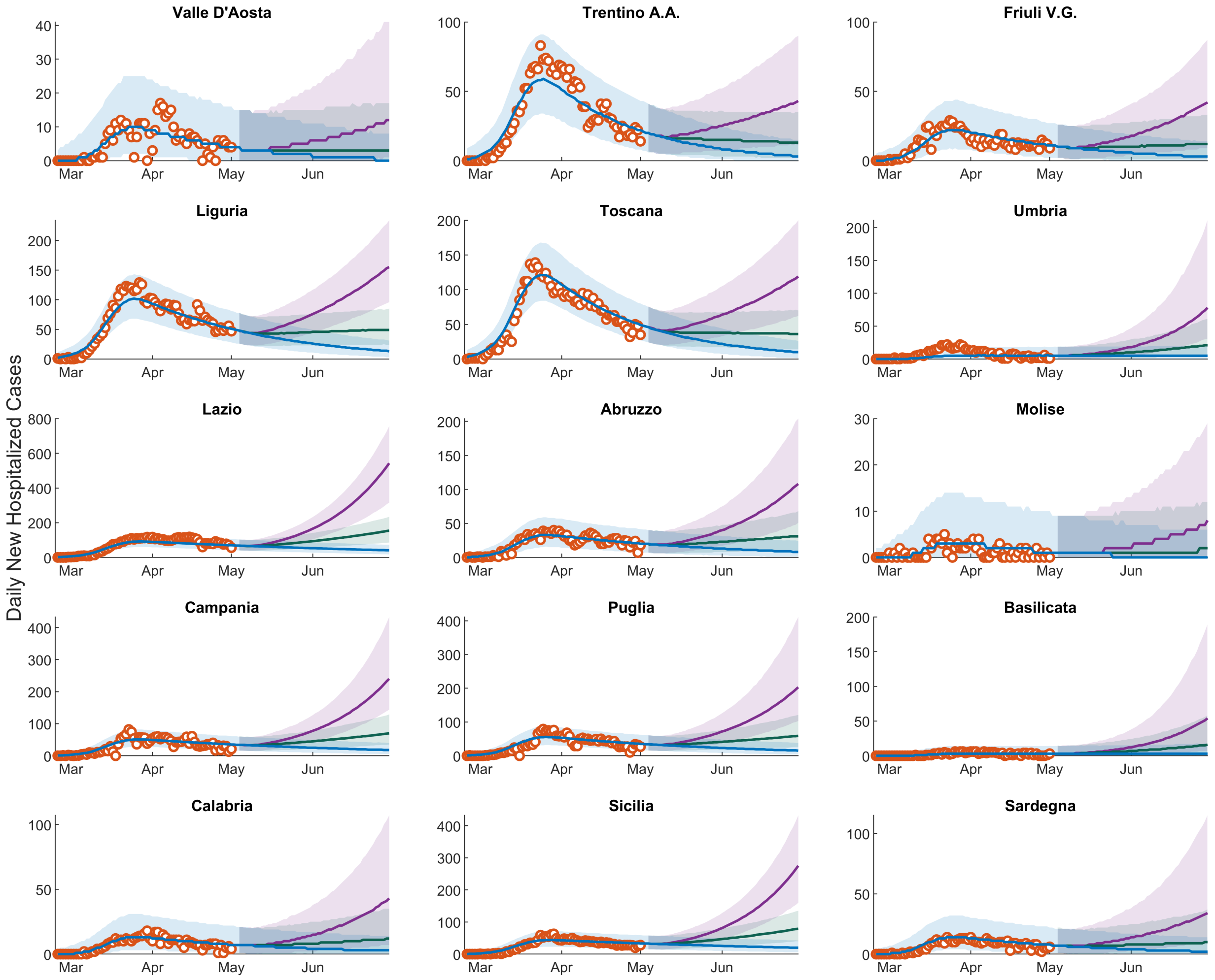

Supplementary Figure 1 Daily numbers of newly hospitalized cases. Shown here are reconstructed data (empty circles), and model results (solid lines and confidence intervals) for the regions not shown in Fig. 2 of the main text. The blue solid line represents the baseline scenario, i.e. the median of the computed results with transmission estimated during lockdown maintained indefinitely beyond May 3, 2020. The green and purple solid lines represent the scenarios corresponding to a release of the containment measures determining an effective increase in the overall transmission rates of respectively 20% and 40%. The 95% confidence intervals are color-coded in analogy to their median scenarios. Plots refer to a fraction of infections leading to heavy symptoms σ equal to 25%.

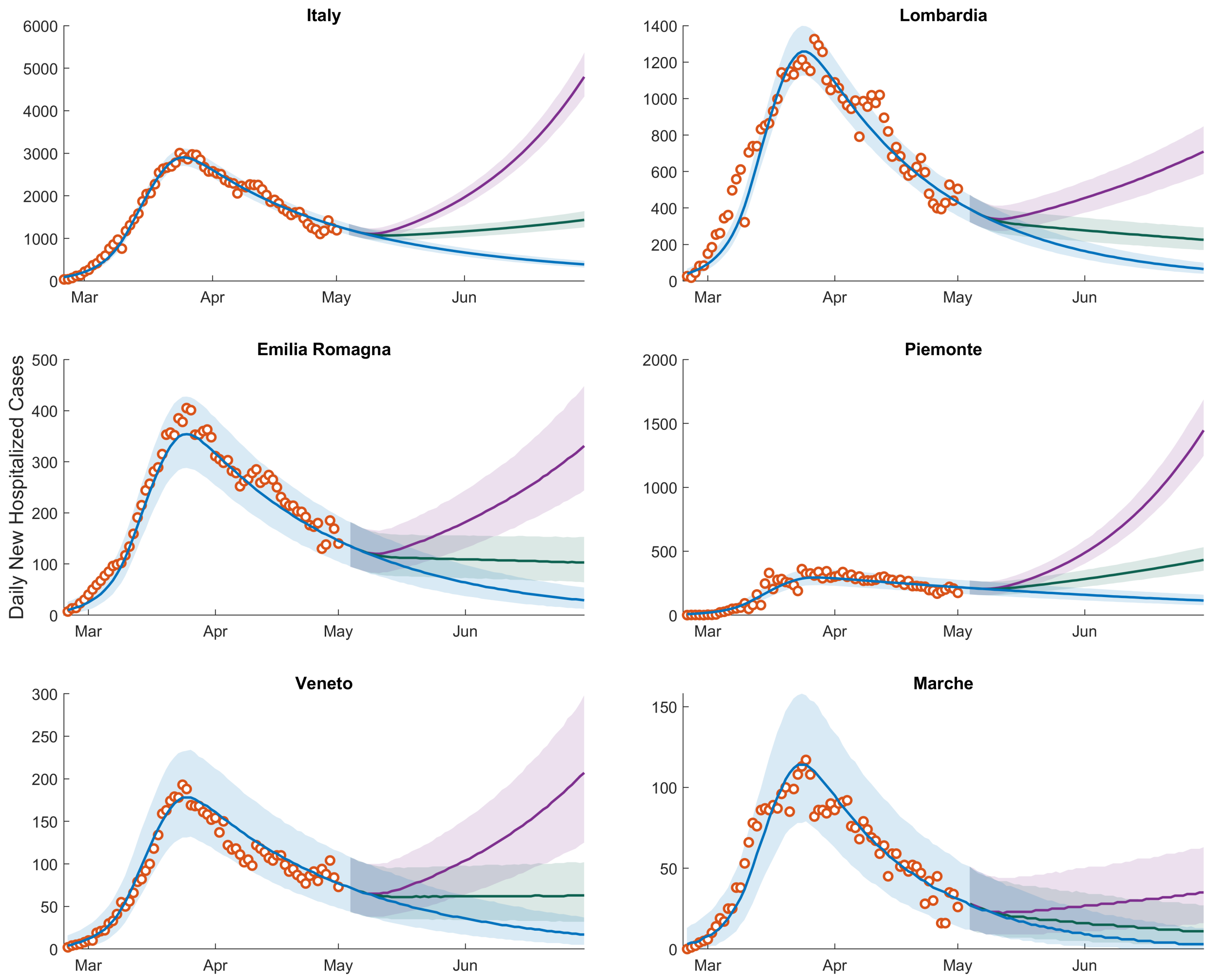

Supplementary Figure 2 Daily numbers of newly hospitalized cases for Italy and its hardest hit regions. Shown here are reconstructed data (empty circles), and model results (solid lines and confidence intervals). Clockwise from top: Italy, Lombardia, Piemonte, Marche, Veneto and Emilia Romagna. The blue solid line represents the baseline scenario, i.e. the median of the computed results with transmission estimated during lockdown maintained indefinitely beyond May 3, 2020. The green and purple solid lines represent the scenarios corresponding to a release of the containment measures determining an effective increase in the overall transmission rates of respectively 20% and 40%. The 95% confidence intervals are color-coded in analogy to their median scenarios. Plots refer to a fraction of infections leading to heavy symptoms σ equal to 50%.

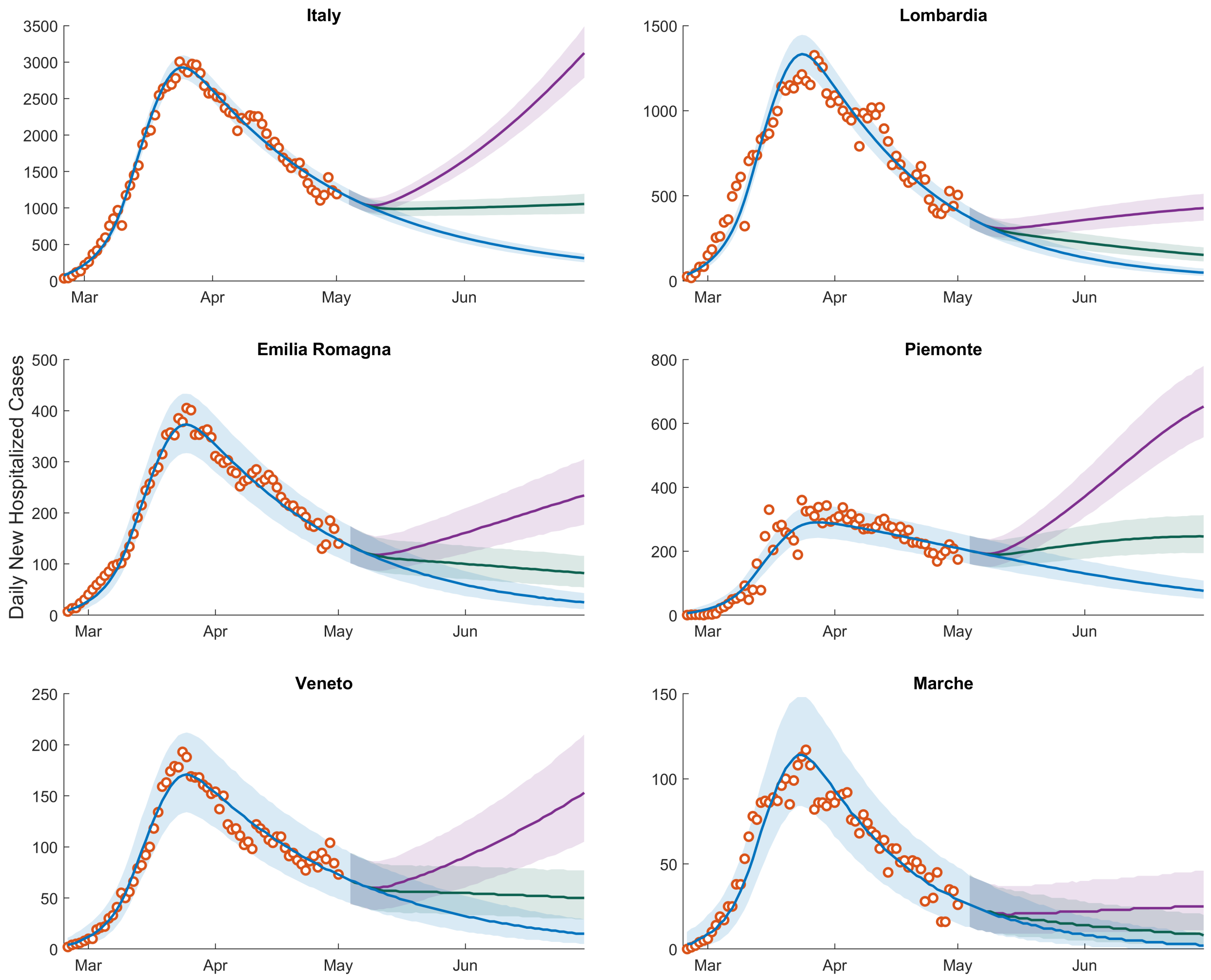

Supplementary Figure 3 Daily numbers of newly hospitalized cases for Italy and its hardest hit regions. Shown here are reconstructed data (empty circles), and model results (solid lines and confidence intervals). Clockwise from top: Italy, Lombardia, Piemonte, Marche, Veneto and Emilia Romagna. The blue solid line represents the baseline scenario, i.e. the median of the computed results with transmission estimated during lockdown maintained indefinitely beyond May 3, 2020. The green and purple solid lines represent the scenarios corresponding to a release of the containment measures determining an effective increase in the overall transmission rates of respectively 20% and 40%. The 95% confidence intervals are color-coded in analogy to their median scenarios. Plots refer to a fraction of infections leading to heavy symptoms σ equal to 10%.

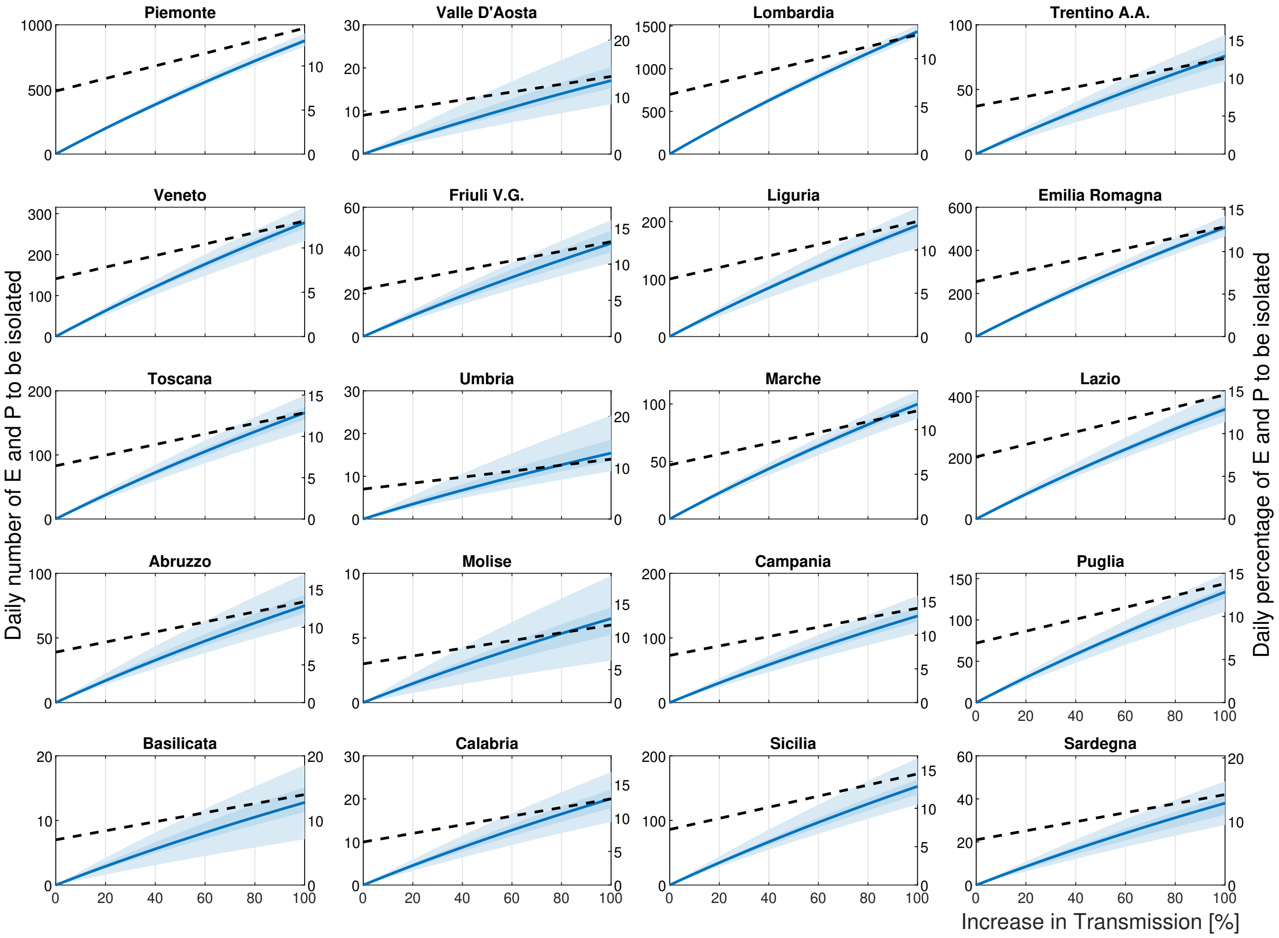

Supplementary Figure 4 Isolation effort. Daily number (left axis) and daily percentage (right axis) of exposed E and individuals at peak infectivity, P, to be isolated daily to maintain the epidemic trajectory onto the blue curve in Fig. 2 of the main text (corresponding to the baseline scenario) despite the possible increase in transmission induced by the actual release of restrictions (horizontal axes). Solid lines refer to median values, shaded areas the 95% (lighter shade) and 50% confidence intervals. The dashed black lines indicate the estimated number of E and P individuals that can be isolated by tracing all the infections generated by the new daily symptomatic cases. All values refer to the case $\sigma = 50\%$.

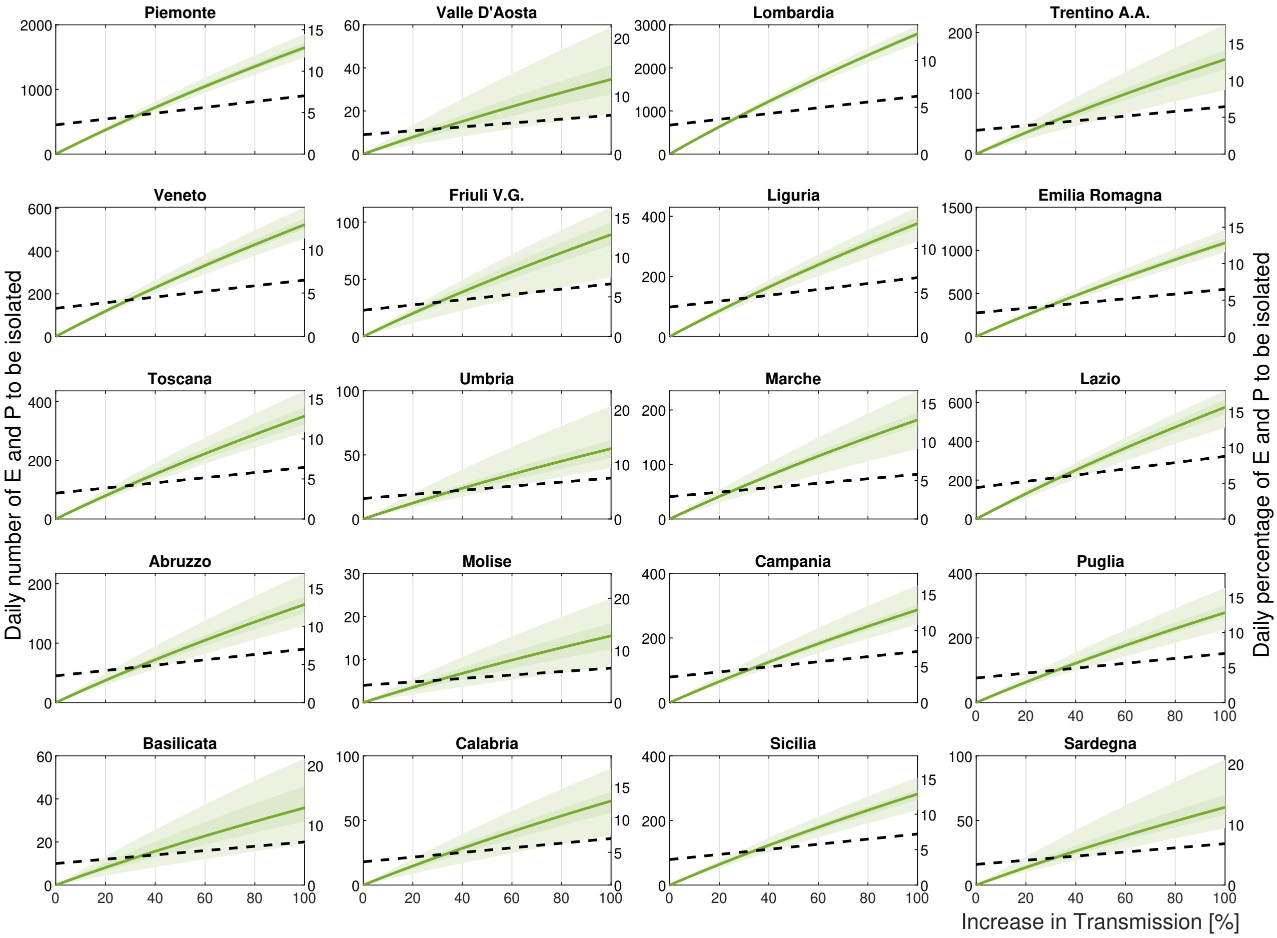

Supplementary Figure 5 Isolation effort. Daily number (left axis) and daily percentage (right axis) of exposed E and individuals at peak infectivity, P, to be isolated daily to maintain the epidemic trajectory onto the blue curve in Fig. 2 of the main text (corresponding to the baseline scenario) despite the possible increase in transmission induced by the actual release of restrictions (horizontal axes). Solid lines refer to median values, shaded areas the 95% (lighter shade) and 50% confidence intervals. The dashed black lines indicate the estimated number of E and P individuals that can be isolated by tracing all the infections generated by the new daily symptomatic cases. All values refer to the case $\sigma = 25\%$.

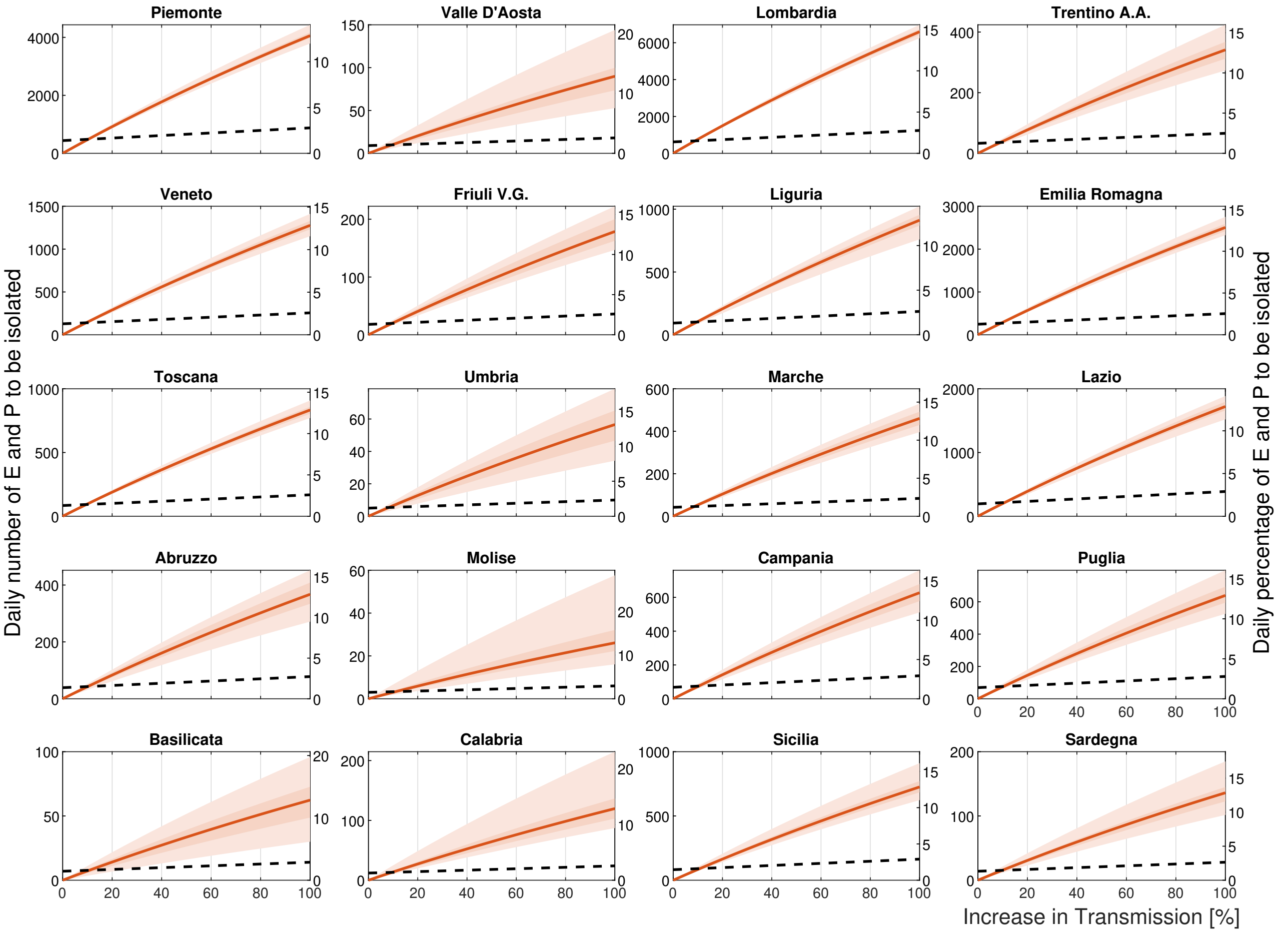

Supplementary Figure 6 Isolation effort. Daily number (left axis) and daily percentage (right axis) of exposed E and individuals at peak infectivity, P, to be isolated daily to maintain the epidemic trajectory onto the blue curve in Fig. 2 of the main text (corresponding to the baseline scenario) despite the possible increase in transmission induced by the actual release of restrictions (horizontal axes). Solid lines refer to median values, shaded areas the 95% (lighter shade) and 50% confidence intervals. The dashed black lines indicate the estimated number of E and P individuals that can be isolated by tracing all the infections generated by the new daily symptomatic cases. All values refer to the case $\sigma = 10\%$.

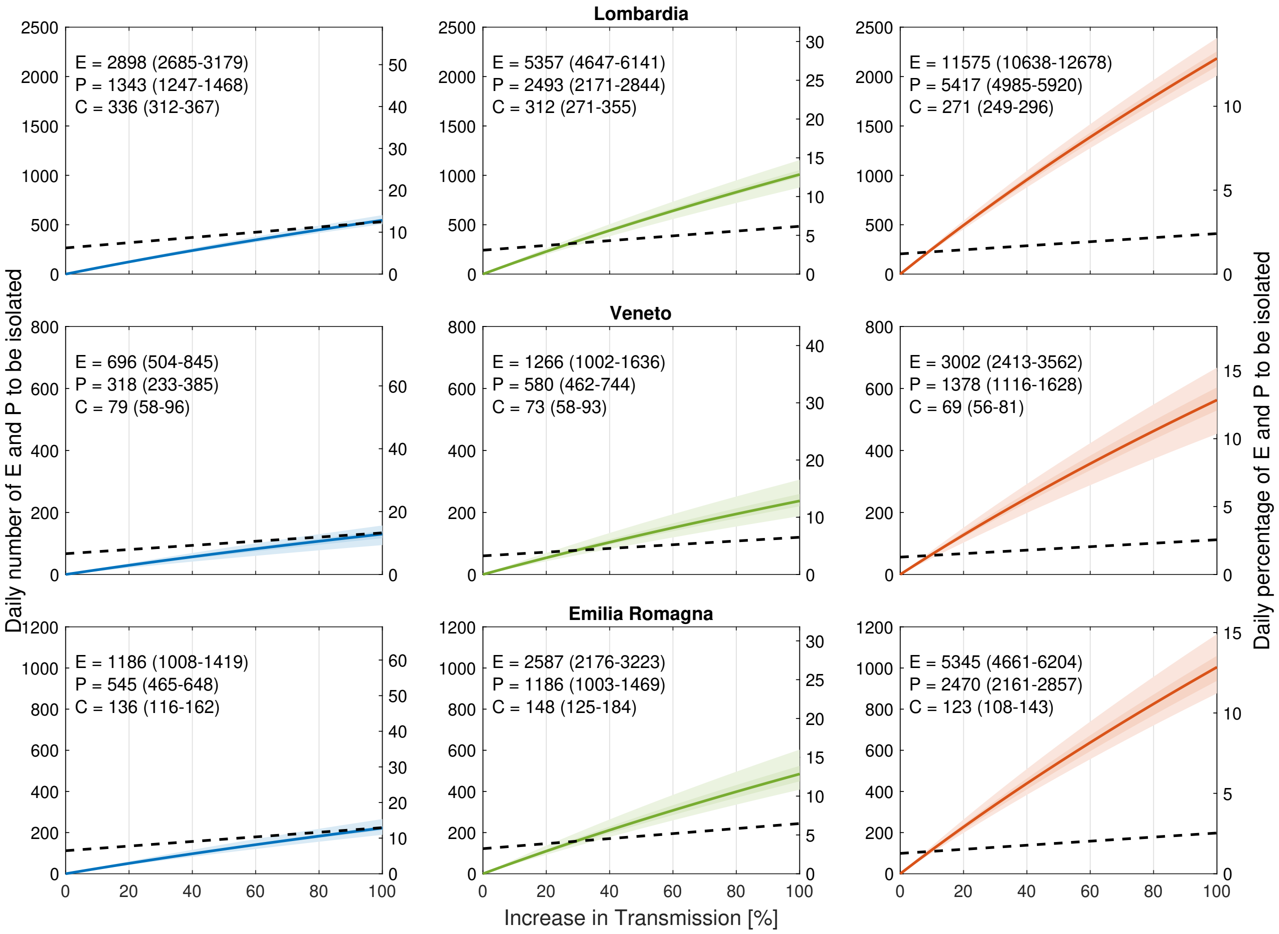

Supplementary Figure 7 Isolation efforts for a delayed lock-down release. Daily number (left axis) and daily percentage (right axis) of exposed E and individuals at peak infectivity, P, to be isolated daily to maintain the epidemic trajectory onto the blue curve in Fig. 2 of the main text (corresponding to the baseline scenario) despite the possible increase in transmission induced by the actual release of restrictions (horizontal axes). Differently from Fig. 4 of the main text, values here refer to a starting date of the relaxation of the restrictive measures delayed by one month (June 4). Different columns refer to different values of the symptomatic fraction σ: 50% (left, blue), 25% (center, green) and 10% (right, red). Solid lines refer to median values, shaded areas to the 95% (lighter shade) and 50% confidence intervals. In each panel, median and 95% confidence interval of E, P and new daily symptomatic cases (C) are given (estimates refer to June 4). The dashed black lines indicate the estimated number of E and P individuals that can be isolated by tracing all the infections generated by the new daily symptomatic cases.

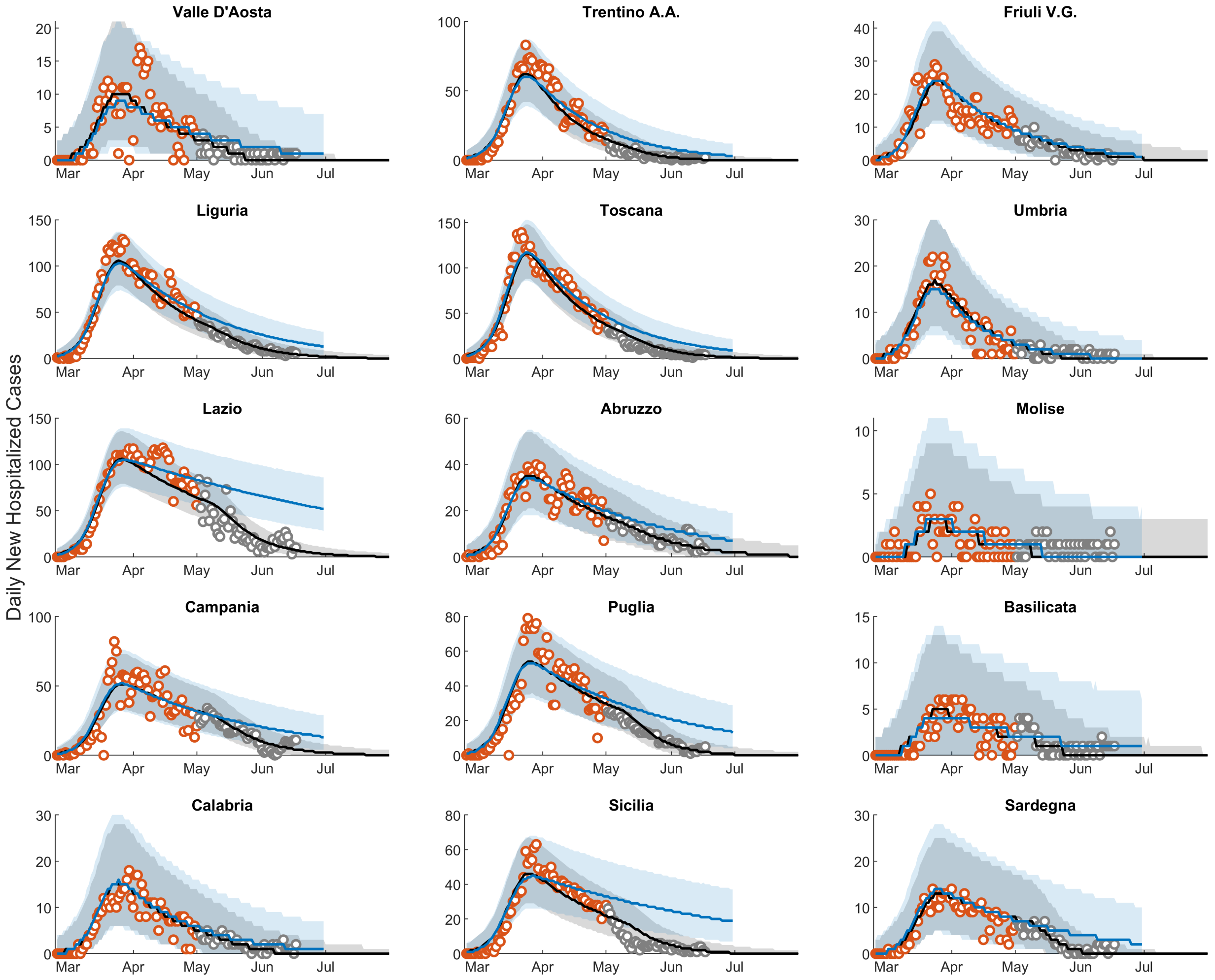

Supplementary Figure 8 Ex-post assessment. Daily numbers of newly hospitalized cases for the regions not shown in Fig. 5 of the main text. Red empty circles represent data available for the projection of the scenarios presented in Supplementary Fig. 1, gray empty circles show the newly available data. Blue colors (solid line: median results, shaded area: 95% confidence intervals) report here, for the sake of comparison, the baseline scenario already presented in Supplementary Fig. 1. Black colors show the results of the updated parameter estimation that exploits all data available up to June 17.

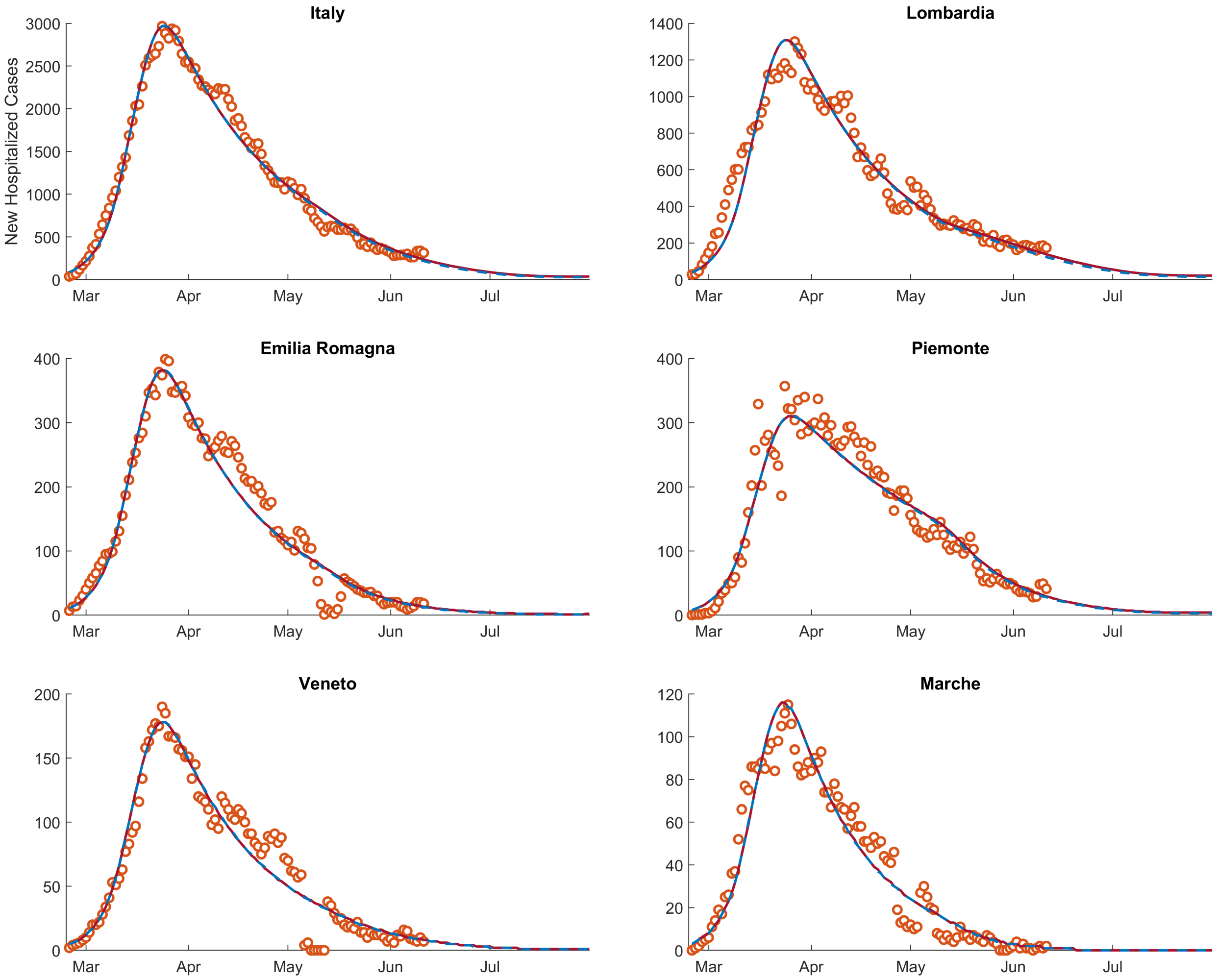

Supplementary Figure 9 Effect of immunity loss. Daily numbers of newly hospitalized cases for Italy and its hardest hit regions. Shown here are reconstructed data (empty circles), and model results (solid lines). Clockwise from top: Italy, Lombardia, Piemonte, Marche, Veneto and Emilia Romagna. Blue dash lines show median results of the simulations reported also in Fig. 5 of the main text. Reddish solid lines show the median of the same simulations save for assuming an immunity duration of three months.
